# New Data on *Boletaceae* (*Agaricomycetes*, *Basidiomycota*) from Central Vietnam with Description of Two New Species and Creation of a New Combination Based on Morphological and Phylogenetic Evidence

**DOI:** 10.3390/jof10030223

**Published:** 2024-03-19

**Authors:** Thi Ha Giang Pham, Eugene Popov, Alina Alexandrova, Daria Ivanova, Olga Morozova

**Affiliations:** 1 Joint Vietnam-Russia Tropical Science and Technology Research Centre, Nguyen Van Huyen, Nghia Do, Cau Giay, Hanoi 122100, Vietnam; 2 Komarov Botanical Institute of the Russian Academy of Sciences, 197376, 2 Prof. Popov Str., 197022 Saint Petersburg, Russia; epopov@binran.ru (E.P.); divanova@binran.ru (D.I.); 3 Faculty of Biology, Lomonosov Moscow State University, Leninskie Gory Str., 1, 12, 119234 Moscow, Russia; alina-alex2011@yandex.ru

**Keywords:** taxonomy, boletoid fungi, molecular phylogeny, new species

## Abstract

Two new species of *Boletaceae* (*Hortiboletus rubroreticulatus* and *Tylopilus aurantiovulpinus*) discovered during an investigation of the mycobiota of Central Vietnam (Kon Chu Rang Nature Reserve; Ta Dung National Park; Bidoup—Nui Ba National Park; Kon Ka Kinh National Park) are described on the basis of molecular and morphological data. Illustrated descriptions of their macro- and microscopic features and discussion on similar taxa are given. Additionally, eight species which were recorded for the first time in Vietnam are listed and illustrated here. A new combination *Kgaria virescens* was made for one of these species. These results were confirmed by the phylogenetic analysis based on nrITS1-5.8S-ITS2, nrLSU, and *tef1α* regions.

## 1. Introduction

Boletoid fungi, i.e., representatives of the ordo *Boletales* characterized by soft putrescent pileate basidiomata with tubulate or rarely lamellate hymenophore [1], play a significant role in tropical ecosystems. As ectomycorrhizal partners of the main forest tree species, including those of *Dipterocarpaceae*, *Fabaceae*, *Fagaceae*, and *Pinaceae*, boletoid fungi improve their nutrition and growth, increase resistance to adverse environmental conditions, as well as participate in the decomposition of organic matter. Many of them are important as a source of nutrition for humans and as potential producers of biologically active substances for medical purposes.

The aim of the present study is to contribute to the knowledge on the fungal diversity of the Dalat and Kon Tum Plateaus, and Ta Dung Ridge (Central Vietnam), where large stands of montane and pedemontane forests have been preserved. While mycological research on the Dalat Plateau began in the first decade of the 20th century (see reviews [2,3]), the first information about the diversity of pileate fungi on the Kon Tum Plateau and Ta Dung Ridge appeared relatively recently [4,5,6,7,8,9,10,11,12,13].

To date, 87 species of boletoid fungi have been cited in literary sources from the territory of Vietnam [3,9,14,15,16,17,18,19,20], including 11 species described as new to science [21,22,23,24,25,26,27,28,29]. However, many earlier records require confirmation since they are given under names based on types from Europe or North America.

## 2. Materials and Methods

### 2.1. Collecting and Site Description

The present study is based on materials collected in four protected areas of the Central Highlands of Vietnam (between 11.8° N and 14.5° N), namely Kon Chu Rang Nature Reserve, Kon Ka Kinh National Park, Ta Dung National Park, and Bidoup—Nui Ba National Park during the expeditions of the Joint Vietnam–Russia Tropical Science and Technology Research Centre (VRTC). All these territories have hilly-to-mountainous terrain with an average height ranging from 600 to 1800 m a.s.l. The highest peaks of the Bidoup—Nui Ba National Park exceed 2000 m a.s.l. The tropical highland climate experiences the effects of the annual monsoon, resulting in a hot and wet summer and a relatively cool and dry winter. The average annual air temperature is 18–22 °C and the average annual rainfall is 1800–2300 mm, while in the highlands it can reach 2800–3000 mm. The main forest types in the reserves are the subtropical, humid, middle-to-high mountain evergreen broad-leaved and mixed forests with representatives of plant species from the families of *Fagaceae* (*Lithocarpus* spp., *Quercus* spp., *Castanopsis* spp.), *Lauraceae*, *Theaceae*, *Fabaceae*, *Clusiaceae*, *Myrtaceae*, *Ericaceae*, *Dilleniaceae*, *Myristicaceae*, *Burseraceae*, *Magnoliaceae*, *Pinaceae* (*Pinus dalatensis, P. kesiya, P. krempfii*), *Podocarpaceae* (*Dacrycarpus imbricatus, Dacrydium elatum*), and *Cupressaceae* (*Fokienia hodginsii*) [30,31,32].

Specimens were photographed in the field, and their macromorphological characters, such as the size, color, shape, and surface of all parts of the basidiomata as well as odor, were documented before drying. The color codes refer to Kornerup and Wanscher [33]. The GPS coordinates of the collection site, habitat, and substrate type were also documented for each collection. Specimens were then dried either in airtight plastic containers with silica gel, or with an electric dryer at a temperature ca. 50 °C, placed on a piece of absorbent paper and packed in plastic Ziploc bags with small amounts of silica gel.

### 2.2. Morphological Study

Microscopic measurements and drawings were made with an Axio Imager. A1 ZEISS equipped with Axiocam 506 color (Carl Zeiss) and an AxioScope A1 light microscope was equipped with a Zeiss AxioCam 1Cc3 digital camera with AxioVisionRel.4.6 software (CarlZeiss, Jena, Saxe-Weimar-Eisenach, Germany). Spores, basidia, and cystidia were observed in squash preparations of small parts of the lamellae or tubes in 5% KOH or 1% Congo Red in concentrated NH_4_OH. The pileipellis was examined from a radial section of the pileus in 5% KOH or 1% Congo Red in concentrated NH_4_OH. Basidiospore dimensions were based on 20 spores and cystidia and basidia dimensions on at least 10 structures per collection. Basidia were measured without sterigmata and the spores without an apiculus. Spore length to width ratios were reported as Q. The dried specimens were deposited in the Mycological Herbarium of the Komarov Botanical Institute RAS (LE) and in the Herbarium of the Joint Vietnam–Russia Tropical Science and Technology Research Centre, Hanoi (VRTC).

### 2.3. DNA Extraction, Amplification, and Sequencing

PCR products were obtained without a DNA purification step using the Thermo Scientific Phire Tissue Direct PCR Master Mix (Thermo Fisher Scientific, Inc., Waltham, MA, USA) standard protocol. The ribosomal ITS1–5.8S–ITS2 region was amplified with the fungal specific primers ITS1F and ITS4B [34]. Sequences of nrLSU-rDNA were generated using the JS1–LR5 pair of primers [35]. Pairs of primers EF1-983F–EF1-1567R and EF1-B-F1–EF1-B-R were used to amplify approximately 500–700 bp of *tef1α* [36,37]. For ITS, PCR was carried out under the following cycling parameters: initial denaturation at 98 °C for 4 min; followed by 35 cycles at 98 °C for 1 min, 52 °C for 1 min, and 72 °C for 1 min; and a final extension at 72 °C for 3 min. For nrLSU, the process was as follows: initial denaturing at 98 °C for 5 min; then 12 cycles of denaturing at 98 °C for 5 s, annealing at 67 °C for 1 min, extension at 72 °C for 1.5 min; then 35 cycles of denaturing at 98 °C for 5 s, annealing at 56 °C for 1 min, extension at 72 °C for 1.5 min; and a final extension step of 72 °C for 10 min. For *tef1a* the process was as follows: initial denaturing at 98 °C for 5 min; then 8 cycles of denaturing at 98 °C for 5 s, annealing at 60 °C for 40 s, extension at 72 °C for 2 min; then 36 cycles of denaturing at 98 °C for 5 s, annealing at 53 °C for 1.5 min, extension at 72 °C for 2 min; and a final extension step at 72 °C for 10 min.

PCR products were visualized using agarose gel electrophoresis and Gel Red staining and subsequently purified with the Fermentas Genomic DNA Purification Kit (Thermo Fisher Scientific Inc., Waltham, MA, USA). Sequencing was performed with an ABI model 3500 Genetic Analyzer (Applied Biosystems, Inc., Waltham, CA, USA).

This work was carried out using equipment from the Core Facility Centre ‘Cell and Molecular Technologies in Plant Science’ of the Komarov Botanical Institute (St. Petersburg, Russia) and of the Joint Vietnam–Russia Tropical Science and Technology Research Centre (Hanoi, Vietnam). Raw data were edited and assembled in MEGA X [38]. Newly generated sequences have been deposited in GenBank.

### 2.4. Alignment and Phylogenetic Analyses

For this study, 12 nrITS, 9 *tef1α*, and 10 nrLSU sequences were newly generated. In addition, 38 nrITS, 20 *tef1α*, and 43 nrLSU sequences, including outgroups, were retrieved from the GenBank database (www.ncbi.nlm.nih.gov//genbank/, accessed on 5 February 2024), using the BLASTn application (https://blast.ncbi.nlm.nih.gov/Blast.cgi accessed on 5 February 2024). The information on all these sequences is presented in Table 1.

Three datasets were analyzed: *tef1α*, nrITS, and nrLSU. DNA sequences were aligned with the MAFFT v.7.110 web tool [73], using the G-INS-i option, and then manually modified where necessary in MEGA X [38]. To determine the phylogenetic positions of the studied collections, phylogenetic reconstructions were performed for three datasets using Bayesian Inference (BI) and Maximum Likelihood (ML) analysis. BI was performed using MrBayes 3.2.1 [74], under a GTR model for *tef1α* and the nrLSU dataset and a GTR + G model for the nrITS dataset. The analyses were run with two parallel searches, four chains, five million generations for ITS, and four million generations for LSU and *tef1α*, starting with a random tree. The trees were sampled every 100 generations. The runs were terminated and the average standard deviations of split frequencies were 0.003879, 0.018290, and 0.002999, respectively. Tracer v1.7.2 [75] was used to check for the convergence of MCMC analyses and to obtain estimates of the posterior distribution of parameter values. ML analyses were run on the IQ-TREE web server [76]. Bootstrap values were obtained using UFBoot [77] with 1000 bootstrap replicates and 1000 maximum iterations. The phylogenetic trees were visualized in FigTree v1.4.4 and edited in Adobe Illustrator CS4. Posterior probability (PP) ≥ 0.95 and bootstrap support (BS) ≥ 65% values are considered significant.

To identify and to reveal new species, we followed both a morphological and phylogenetic species concept, referring to the examples for fungi in Taylor et al. [78]. Monophyletic clades are recognized as phylogenetic species when they are supported by the received phylogenetic trees and when ITS sequence similarities are less than 97%.

## 3. Results

### 3.1. Phylogenetic Analysis

Over the course of data analysis, it became clear that constructing multigene trees using an incomplete dataset for each species can lead to errors that are not obvious in the results of such analysis. Therefore, in cases where the tree topology differs for different markers, we prefer to analyze each marker separately. The overall topologies of the BI and ML trees were nearly identical for all datasets. Therefore, we present a single phylogram for each marker.

The full *tef1α* dataset contained 50 sequences with 660 characters (gaps included). *Gyroporus cyanescens* and *Gyrodon lividus* were selected as outgroups. Thirteen sequences were newly generated. The dataset also includes 37 other sequences retrieved from the GenBank NCBI database—the closest possible representatives of the *Boletaceae*.

The full nrITS dataset contained 52 sequences with 783 characters (gaps included). *Gyroporus cyanescens* and *G. castaneus* were selected as the outgroup. Besides the 16 newly generated sequences, the dataset includes 36 other sequences retrieved from the GenBank NCBI database—the closest possible representatives of the *Boletaceae* genera.

The full nrLSU dataset contained 58 sequences with 891 characters (gaps included). *Gyrodon lividus* and *Gyroporus cyanescens* were chosen as an outgroup. The dataset contained 13 newly generated sequences, as well as 45 representatives of the main subdivisions of the *Boletaceae*.

**Figure 1:**
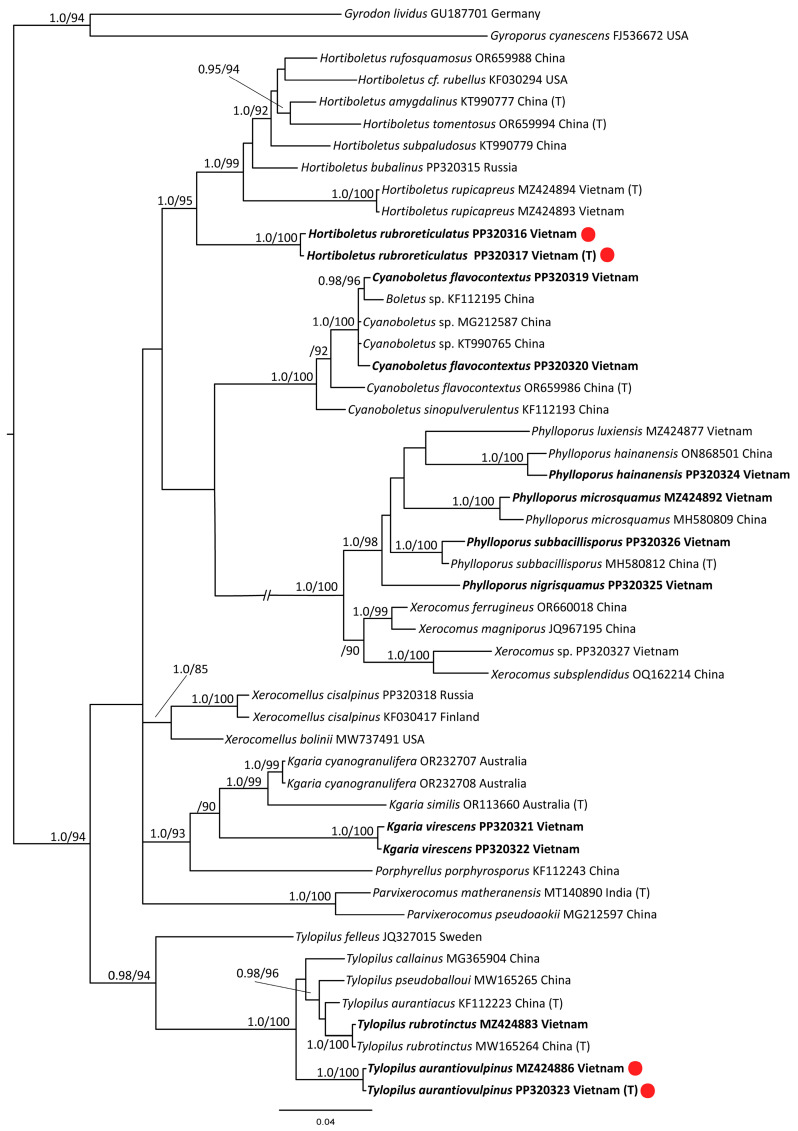
Phylogenetic tree derived from Bayesian Inference (BI), based on *tef1α* data. Values of the posterior probability (PP ≥ 0.95) from the BI and of the bootstrap support (BS ≥ 65) from the ML analysis are given to the left of the nodes. The scale bar represents the number of nucleotide changes per site. New species and new records are in bold. T—holotype. New species are marked with a red circle.

**Figure 2:**
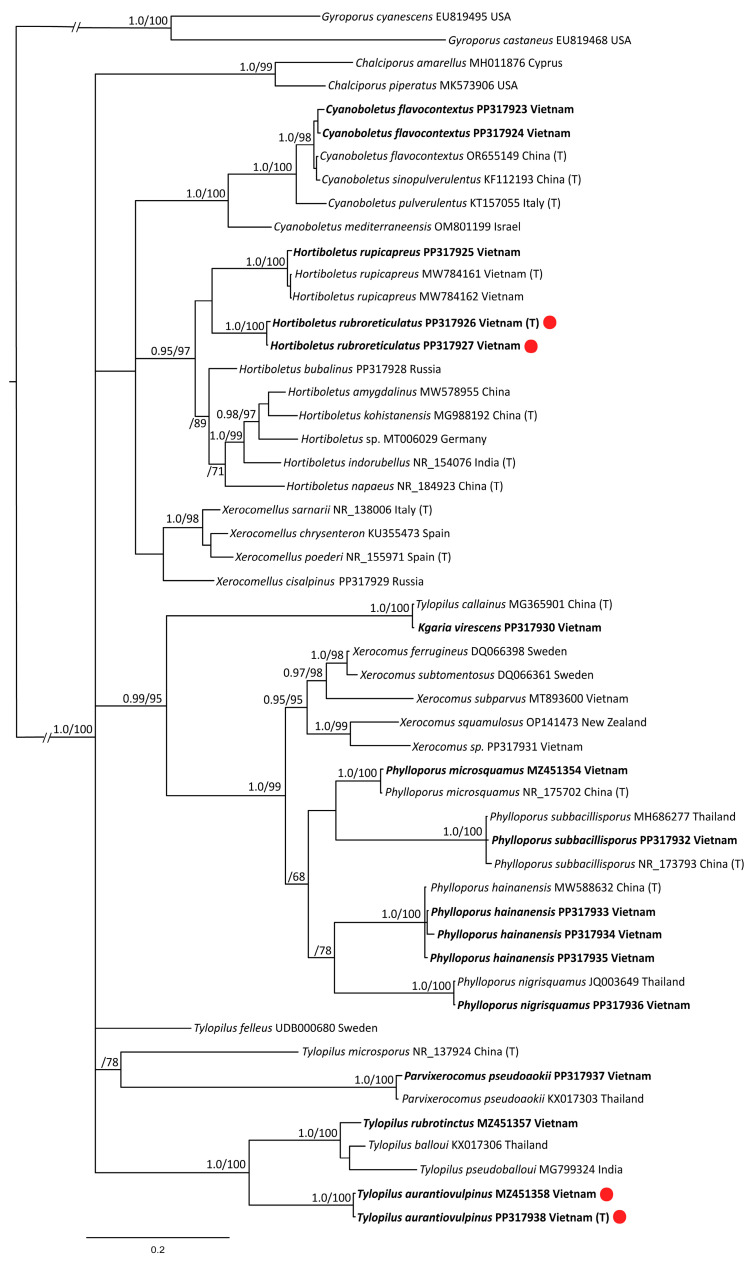
Phylogenetic tree derived from Bayesian Inference (BI), based on nrITS1-5.8S-ITS2 region data. Values of the posterior probability (PP ≥ 0.95) from the BI and of the bootstrap support (BS ≥ 65) from the ML analysis are given to the left of the nodes. The scale bar represents the number of nucleotide changes per site. New species and new records are in bold. T—holotype. New species are marked with a red circle.

**Figure 3:**
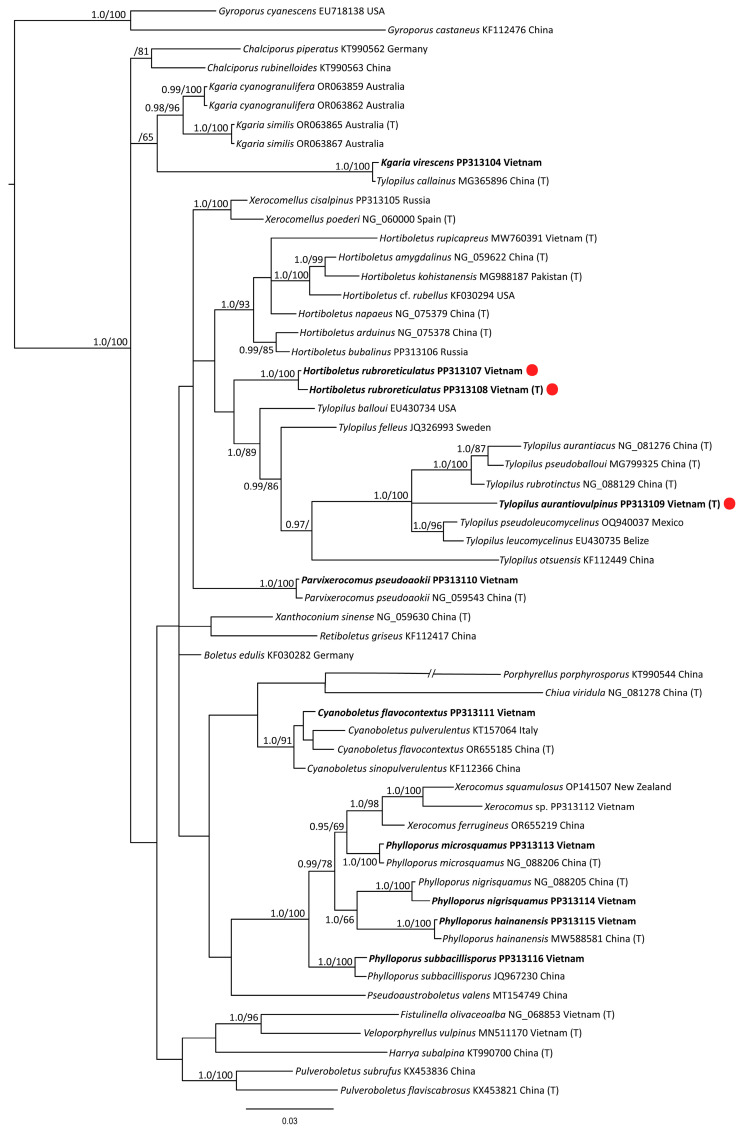
Phylogenetic tree derived from Bayesian Inference (BI), based on nrLSU data. Values of the posterior probability (PP ≥ 0.95) from the BI and of the bootstrap support (BS ≥ 65) from the ML analysis are given to the left of the nodes. The scale bar represents the number of nucleotide changes per site. New species and new records for are in bold. T—holotype. New species are marked with a red circle.

The results of the phylogenetic analyses are presented in Figure 1 (*tef1α*), Figure 2 (nrITS), and Figure 3 (nrLSU). The phylogenetic trees demonstrated rather different topology and different support values of clades. Based on the obtained topologies, the taxonomic positions of the newly generated sequences are discussed below.

*Hortiboletus rubroreticulatus.* New species is nested within the /Hortiboletus clade with high support in *tef1α* and ITS trees, but its position is relatively independent in the LSU tree. The latter can be explained by the low resolution of the LSU marker.

*Tylopilus aurantiovulpinus.* New species is nested within the /Tylopillus balloui-complex clade with high support values in all trees.

*Cyanoboletus flavocontextus.* Our molecular phylogenetic analyses indicate the new collections and the holotype of the *Cyanoboletus flavocontextus* group together with a strong statistical support in all three trees. However, while in the ITS tree, these sequences are almost identical; in *tef1α* and LSU trees, they slightly differ but not enough to consider them as separate species.

*Kgaria virescens* (=*Tylopilus virescens*; =*T. callainus*). In the phylogenetic analysis, the species is represented by our two sequences. The specimens were collected in the Kon Chu Rang Nature Reserve with an interval of 7 years—in 2016 and 2023. Despite the existing morphological differences, these sequences are almost identical. They are also identical to the ITS and LSU sequences of the holotype specimen of *Tylopilus callainus*, which is recognized as a synonym of *T. virescens* [79]. However, there is no correlation between our *tef1α* sequences and the only *tef1α* sequence of *Tylopilus callainus* presented in GenBank (MG365904) and cited in relevant publications [61,64]. The sequence MG365904 nests in a clade with representatives of the *T. balloui* complex. It is also present in the multigene tree in the work devoted to *Tylopilus* of China [61]. Since this sequence is not from the holotype and it is rather short, while our ITS and LSU sequences in double repetition coincide with the ones from the holotype, we suspect that an error, perhaps some kind of contamination, could have crept in here. Our data show that the sequences of this species do not form a common clade with other representatives of the genus *Tylopilus* but occupy a position isolated from them. In the *tef1α* tree, they are grouped together with *Porphyrellus* and *Kgaria* with high statistical support. There are no ITS data on the genus *Kgaria* in the GenBank. In this case, the grouping of this species with representatives of *Xerocomoidea* raises some doubts. This may be due to the “long branch attraction” in the absence of close sequences. In the LSU tree, our sequences grouped with the /Kgaria-clade but without significant support. Based on these results, the taxonomic position of *Tylopilus virescens* should be considered uncertain but close to the genus *Kgaria*. Until the problem is finally resolved, we propose a new combination *Kgaria virescens*.

*Parvixerocomus pseudoaokii.* The sequences in our collection and the holotype of this species are almost identical in the analysis based on the LSU dataset. In the ITS tree, it is identical to the specimen from Thailand (KX017306).

As a result of phylogenetic analysis, it was possible to confirm the identity of sequences obtained from specimens of the genus *Phylloporus* with the corresponding holotypes of the following species: *Phylloporus hainanensis* (ITS, LSU), *Ph. microsquamus* (ITS, LSU), *Ph. nigrisquamus* (LSU from holotype, ITS from reference specimen from Thailand), and *Ph. subbacillisporus* (ITS, *tef1α*).

*Tylopilus rubrotinctus.* Sequences of our collection and the holotype of this species are identical in the analysis based on the *tef1α* dataset, and they are nested within the /Tylopillus balloui-complex clade.

### 3.2. Taxonomy

#### 3.2.1. New Species

***Hortiboletus*** Simonini, Vizzini & Gelardi, Index Fungorum 244: 1 (2015).

*Hortiboletus* was established to accommodate *Boletus rubellus* Krombh. and allied species with smooth spores as opposed to the closely related genus *Xerocomellus* that includes species with striate spores. The species were also characterized by the common occurrence of small red dots in the context at the stipe base, the average spore quotient (Qm) lower than 2.5, and its habitat in the park, gardens, and other ruderal places. With the inclusion of several new species, the genus concept expanded, including species without red dots, with much longer and narrower spores, and with a wider ecological range. According to Wu et al. [41], this genus differs from other genera of *Boletaceae* by a combination of the dry and subtomentose pileus usually with a wine-red tinge, a yellow–brown to red–brown stipe without a purplish tinge, and a palisadoderm pileipellis composed of vertically arranged, more or less broadened, and often incrusted hyphal elements, with the terminal cells reaching almost the same level. Fourteen species are listed in Index Fungorum (https://www.indexfungorum.org, accessed on 10 February 2024). One species (*Hortiboletus rupicapreus* Svetash., A.V. Alexandrova, O.V. Morozova & T.H.G. Pham) from Vietnam was recently described [29].

Type species: *Hortiboletus rubellus* (Krombh.) Simonini et al.

**Figure 4:**
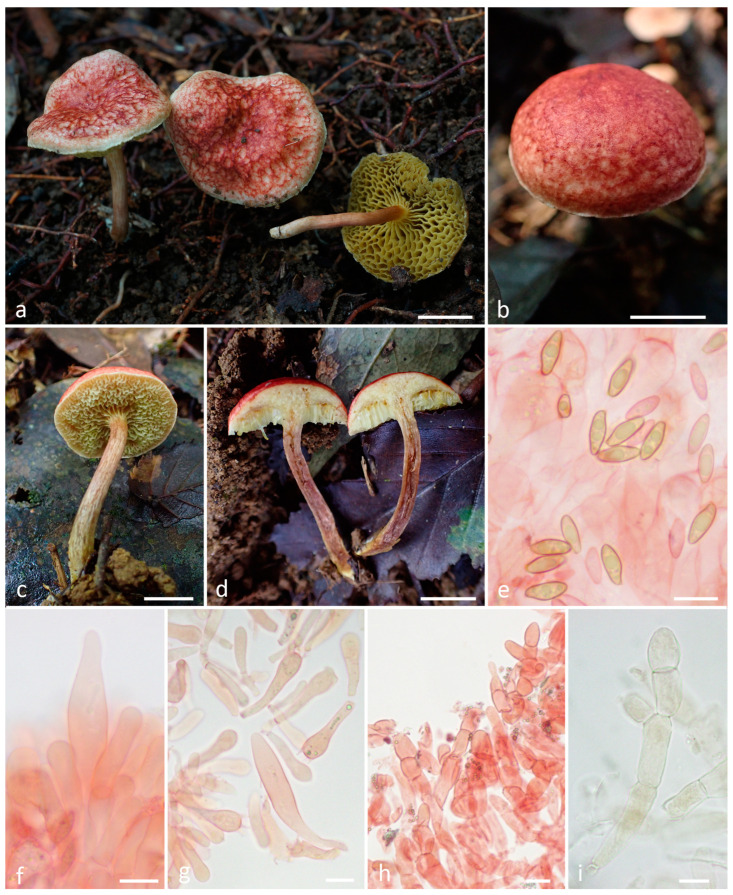
*Hortiboletus rubroreticulatus*: (**a**–**d**) basidiomata; (**e**) basidiospores; (**f**) cheilocystidia; (**g**) basidia and pleurocystidium; (**h**,**i**) pileipellis ((**a**) from LE F-344054, (**b**–**i**) from LE F-344053, holotype). Scale bars (**a**–**d**) 1 cm, (**e**–**i**) 10 μm.

***Hortiboletus rubroreticulatus*** T.H.G. Pham, O.V. Morozova, E.S. Popov, sp. nov. (Figure 4).

MycoBank: MB852382.

*Etymology*. From ruber (Lat.) meaning red and reticulum (Lat.) meaning net, referring to the feature of the pileus surface—a reddish net on a whitish background.

*Holotype*. Vietnam, Lam Dong Province, Lac Duong District, Da Chais Commune, Bidoup—Nui Ba National Park, N 12.186726°, E 108.678356°, 1456 m a.s.l., on soil in mixed forest next to Giang Ly ranger station with a predominance of Fagaceae (*Lithocarpus* sp., *Quercus* sp.), Theaceae (*Schima wallichii*), Magnoliaceae (*Michelia* sp.), Podocarpaceae (*Dacrycarpus imbricatus*), 22 October 2023, col. T.H.G. Pham, LE F-344053. Isotype in VRTC (63HG23).

*Diagnosis*. The species is recognized by a small xerocomoid basidiomata with the pileus covered with a red squamulose reticulum on a whitish background, by elongate fusiform hymenial cystidia and by the long and narrow smooth basidiospores with a Q around three.

*Basidiomata* small-sized, xerocomoid. Pileus 10–25 mm diam; hemispherical to convex when young, then broadly convex to applanate; surface dry or slightly viscid when moist; fibrillose-tomentose to finely squamulose; firstly, almost completely unevenly pinkish-red, greyish-red or brownish-red (9A8, 9B8, 10B5–6, 10C5–6, 11A5–6, 11D6–8), and whitish near the pileus margin; then with the red squamulose reticuloid patterns on the whitish background; context up to 5 mm thick, light yellow, greyish yellow (4B8, 4C6–8), unchanging in color. *Hymenophore* tubulate, adnate to sinuate, sometimes depressed around the stipe; surface pale yellow at a young age (2A3), greyish-yellow with age (2B3–4), unchanging when exposed; tubes concolorous with hymenophore surface, 3–6 mm long; pores angular or subround, 1–2 mm diam. *Stipe* 20–45 × 3–6 mm, central, cylindrical, equal or slightly enlarged downward and tapering towards the base; solid; longitudinally fibrillose; yellowish-brown, brownish red (9C5), basal part yellowish-brown (5D4-5); context greyish red, brownish-vinaceous (9E5-6, 12C4-6, 12D4-6), light yellow in upper part, unchanging in color when exposed. Basal mycelium yellowish. Odor and taste are not recorded.

*Basidia* 34–41 × 9.5–10.8 μm, clavate, 4-spored. *Basidiospores* (11–)11.5–12.5(–13) × 4–4.6 μm, on average 12 × 4.3, Q = 2.6–3.2, Qav = 2.8, subfusiform to fusiform in side view with slight suprahilar depression, subfusoid in face view, pale yellow to yellowish brown in 5% KOH, smooth. *Hymenophoral trama* intermediate, subregular to divergent, of cylindrical hyphae, 5–8 μm wide. Edge of tubes heterogeneous. *Cheilocystidia* 50–75 × 9–12 μm, narrowly fusiform, fusoid-ventricose or lageniform, hyaline or pale yellow in water or 5% KOH, thin-walled, smooth. *Pleurocystidia* 51–80 × 9–13 μm, same as cheilocystidia. *Pileipellis* a trichoderm to palisadoderm of vertically arranged or intertwined hyphae, consisting of chains of intermixed both moderately elongate and rather short subglobose elements 12–30 × 7.5–10 μm, with subrounded, ovoid, clavate, usually with rounded apex, sometimes lageniform terminal cells, rarely incrusted. Subpellis consists of elongate to cylindrical hyphae encrusted by a granular pigment, often with “zebra-incrustation” arrangement. *Stipitipellis* a cutis with caulohymenium in upper part; caulocystidia 25–50 × 6–10 μm, subfusiform, narrowly clavate to subcylindric. Clamp connections absent in all tissue.

*Habitat and distribution*. Solitary and in small groups on soil in tropical mountain evergreen forests. Currently known only from Vietnam.

*Additional specimens examined*. Vietnam, Gia Lai Province, K’Bang District, Son Lang Commune, Kon Chu Rang Nature Reserve, N 14.49667°, E 108.56106°, 980 m a.s.l., on soil in middle mountain evergreen mixed forest with a predominance of *Podocarpaceae* (*Dacrydium elatum, Dacrycarpus imbricatus*), *Magnoliaceae*, *Burseraceae* (*Canarium*), *Myrtaceae* (*Syzygium*), 28 May 2016, col. E.S. Popov, LE F-344054.

Notes: *Hortiboletus rubroreticulatus* species occupies an isolated position in the genus *Hortiboletus*, primarily due to the peculiarities of the pileipellis, in which small scales are unevenly arranged into a reticulate pattern on a white background. Some similarities can be observed between new species and species that are characterized by the areolate, cracked at the maturity surface of the pileus, when the scales diverge with age and also show a white background (*H. subpaludosus*, *H. amygdalinus*, *H. kohistanensis*, *H. napaeus*, and *H. rufosquamosus*). However, *H. amygdalinus* lacks reddish tinges in the color of the basidiomata and possesses broader basidiospores [41]. *H. rufosquamosus* also has broader spores [42]. *H. napaeus* has shorter spores [51]. *H. kohistanensis* has large, up to 7.5 cm basidiomata [53].

Another feature unusual for this genus is the slightly sticky surface of the pileus when wet. Perhaps this feature is overestimated since both finds were made during the rainy season.

From a phylogenetic point of view, the position of the species in the tree is rather ambiguous. In the ITS and *tef1α* trees, this species is grouped together with other members of the genus with strong statistical support, albeit in a basal position. But they do not group together with enough support in the LSU tree. Perhaps as more data accumulate, the true taxonomic position of this species will become clearer.

***Tylopilus*** P. Karst., Revue mycol., Toulouse 3(9): 16 (1881).

In a broad sense, *Tylopilus* turned out to be polyphelytic, and based on molecular genetic evidence, in recent years, a significant number of new genera were separated from it. According to the latest generalizations [41,61], the genus *Tylopilus*, in a strict sense, can be recognized by its bitter taste in most species; white to pallid or yellowish context without discoloration or staining red to reddish brown, or blue when hurt; white to cream then pinkish to pink or yellowish on the hymenophoral surface; cutis, trichoderm, hymeniderm, or palisadoderm pileipellis; and smooth basidiospores. In total, 150 of the *Tylopilus* species are accepted in the Index Fungorum (https://www.indexfungorum.org, accessed on 10 February 2024), and 7 species have been reported in Vietnam to date.

Type species: *Tylopilus felleus* (Bull.) P. Karst.

**Figure 5:**
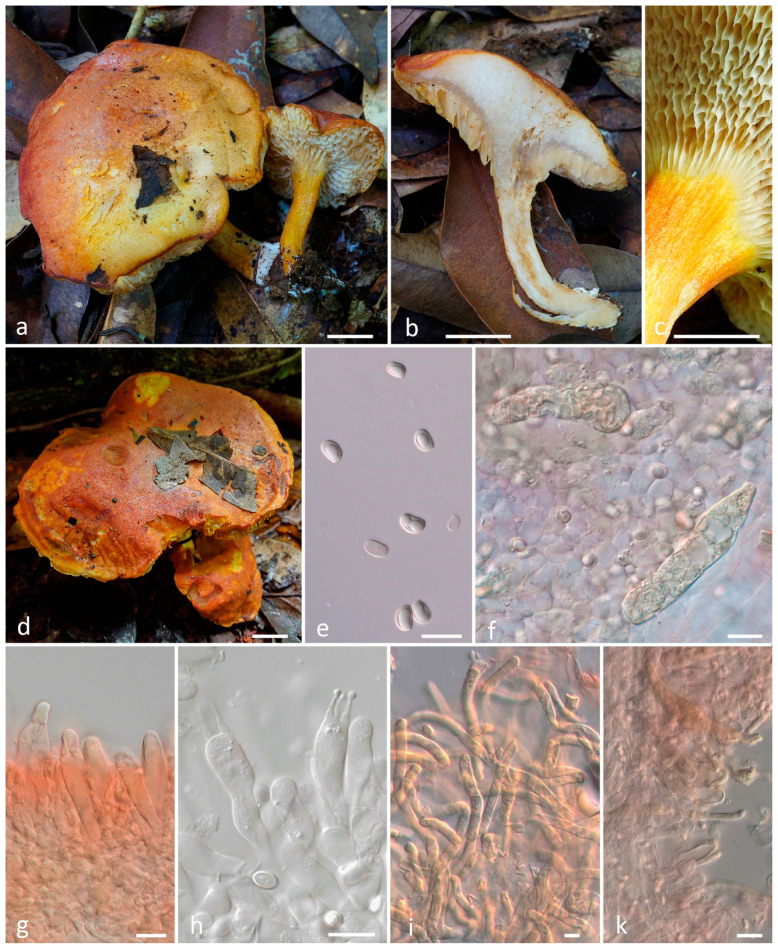
*Tylopilus aurantiovulpinus*: (**a**–**d**) basidiomata; (**e**) basidiospores; (**f**) pleurocystidia; (**g**) cheilocystidia; (**h**) basidia; (**i**) pileipellis; (**k**). stipitipellis ((**a**,**b**,**e**,**f**,**h**,**k**) from LE F-344061, holotype; (**c**,**d**,**g**,**i**) from LE 312700). Scale bars (**a**–**d**) 1 cm, (**e**–**i**,**k**) 10 μm.

***Tylopilus aurantiovulpinus*** A.V. Alexandrova, O.V. Morozova, D.D. Ivanova, T.H.G. Pham, sp. nov. (Figure 5).

MycoBank: MB852383.

*Etymology*. From “aurantius” (Lat.) meaning orange and “vulpis” (Lat.) meaning fox, referring to the bright orange color of the basidiomata.

*Holotype.* Vietnam, Kon Tum Plateau, Gia Lai Province, K’Bang district, Son Lang Commune, Kon Chu Rang Nature Reserve, N 14.49170°, E 108.56621°, 990 m a.s.l., on the soil in a middle-mountain evergreen mixed forest with a predominance of *Podocarpaceae* (*Dacrydium elatum*, *Dacrycarpus imbricatus*), *Magnoliaceae*, *Burseraceae*, *Myrtaceae*, 29 May 2016, col. O.V. Morozova, LE F-344061. Isotype in VRTC 286VN16.

*Diagnosis*. Among other species of the *Tylopilus balloui*-complex with bright orange or brownish-orange basidiomata and rather small phaseoliform to ovoid basidiospores, *Tylopilus aurantiovulpinus* stands out due to the decurrent on the stipe boletinoid hymenophore, consisting of large angled tubes.

*Basidiomata* small to medium-sized. *Pileus* 3–9 cm in diam., at first hemispherical then plano-convex and finally broadly pulvinate-flattened, sometimes slightly depressed at center when mature, regularly to unevenly shaped; margin steady to faintly wavy-lobed, initially curved downwards and finally completely plane to uplifted or even slightly revolute; surface dry or subviscid when wet, matte, finely subtomentose, not cracked; variable in color, ranging from orange-yellow (4B7–8), greyish-orange (5B6-7), brownish-yellow and orange (5C6–8), golden brown (5D7) to cinnamon and light brown (6D6–8), sometimes with bleached or darkened spots and areas. *Context* cream or light yellow (4A4–5) to pale orange (5A2–3), not discolored when bruised or turns slightly yellow-brown. *Hymenophore* boletinoid, adnate when young, distinctly decurrent to stipe when mature; surface firstly cream or pale orange (5A1–3), becoming salmon (6A2) with a pinkish tinge when mature, without discoloration when bruised or only slowly becoming pale dirty-brownish. *Pores* boletinoid, very large, subangular, and radially elongated, with concolorous or yellow edge, up to 3.5 mm wide and 5.5 mm in length; tubes 5–7 mm long. *Stipe* 5–8 × 1–2 cm, subcylindrical, slightly clavate or tapering towards the base, solid, firm, dry, pale orange (5A3) in upper part and light to deep orange (5A4–6) or golden yellow and greyish orange (5B6–7) in the central part, surface smooth to very finely fibrillose, context concolorous to it in the pileus, cream, light yellow to pale orange, unchanging; basal mycelium white. Odor indistinct, mild taste. Spore print yellowish to pinkish.

*Basidia* 25–33 × 6–8 μm, cylindrical, narrowly clavate to clavate, four-, sometimes two-spored, without clamps. *Basidiospores* 5.6–7.7 × 3.5–4.3 μm, on average 6.7 × 3.9, Q = 1.7–2.1, Qav = 1.7, allantoid, phaseoliform, subellipsoid to elongated, apex rounded, with a short apiculus and without a suprahilar depression, smooth, having one or two oil droplets, and hyaline to yellowish in KOH. *Cheilocystidia* 33–47 × 7–13 μm fusiform, narrowly clavate or lageniform, hyaline or with yellow content. *Pleurocystidia* 40–55 × 8–16 μm, abundant, fusiform or lageniform, thin-walled, with pale yellowish to bright golden-yellow oily content, some of them arising from the hymenophoral trama. *Hymenophoral trama* boletoid, divergent. *Pileipellis* a trichoderm, composed of interwoven hyphae 5–9 μm wide with subcylindrical terminal cells, yellowish to pale-brownish in KOH. *Stipitipellis* of slender, parallel to subparallel and longitudinally running, adpressed hyphae, hyaline to pale yellowish in KOH; caulocystidia rare and similar in shape, size, and color to hymenial cystidia. Clamp connections absent.

*Habitat and distribution*. Solitary and in small groups on soil in tropical mountain evergreen forests. Currently known only from Vietnam.

*Additional specimens examined*. Vietnam, Kon Tum Plateau, Gia Lai Province, K’Bang district, Sơn Lang commune, Kon Chu Rang Nature Reserve, N 14.505156°; E 108.581400°, 1000 a.s.l., on soil in the tropical lower mountain deciduous polydominant forest with a predominance of *Lauraceae* (*Litsia*), *Burseraceae* (*Canarium*), *Myrtaceae* (*Syzygium*), *Hamamelidaceae* (*Simingtonia*), and *Fagaceae* (*Lithocarpus*, *Quercus*, *Castanopsis*), 27 May 2016, col. A.V. Alexandrova, LE 312700 (Vn16-141) (in [9] as *Tylopilus* aff. *balloui* 2).

Notes: Morphologically this species belongs to the *Tylopilus balloui*-group characterized by an orange-red color of the pileus, an orange-yellow stipe, a yellowish-pinkish hymenophore, bean-shaped, rounded spores with a length-to-width ratio of less than two, and a pleuro- and cheilocystidia fusiform shape.

The first species of this group *Tylopilus balloui* (Peck) Singer was described by C.H. Peck in 1912 (as a *Boletus*) from North America [80]. The generic recognition of *T. balloui* was ambiguous as an extension of either *Gyrodon*, *Rubinoboletus* and *Gyroporus* [81]; however, phylogenetic studies confidently place it in a core *Tylopilus* clade [63,66,82,83,84]. This species was reported from various regions of the tropical and subtropical areas, and it was considered that *T. balloui* sensu lato has a very wide distribution with some morphological variability for each region.

Molecular genetic methods have made it possible to begin to distinguish individual species within the complex. So, six species from Central America have already been described: *Tylopilus dunensis* Magnago & M.A. Neves; *T. leucomycelinus* (Singer & M.H. Ivory) R. Flores & Simonini; *T. oradivensis* Osmundson & Halling; *T. pseudoleucomycelinus* Ayala-Vasquez, Pinzon & Montoya; *T. pygmaeus* Magnago & R.M.B. Silveira; *T. tropicalis* Montoya, Bandala, Ramos & Halling [67,82,84,85,86,87]. Six species were revealed in Southeast Asia: *T. aurantiacus* Yan C. Li & Zhu L. Yang; *T. griseiviridus* Yan C. Li & Zhu L. Yang; *T. griseolus* Yan C. Li & Zhu L. Yang; *T. rubrotinctus* Yan C. Li & Zhu L. Yang from China [61], *T. pseudoballoui* D. Chakr., K. Das & Vizzini from India [66] (Chakraborty et al., 2018), *T. fuscatus* (Corner) Yan C. Li & Zhu L. Yang from Singapore [81], and *T. phaseolisporus* (T.H. Li, R.N. Hilton & Watling) Osmundson, Bougher, R. Rob. & Halling from Australia [83].

Among these species, *Tylopilus aurantiovulpinus* stands out due to its hymenophore of the boletinoid type, with very large angular and radially elongated pores slightly deccurent to the stipe. Only a yet-undescribed species from Australia has a hymenophore of a similar type [88]. Other species of *Tylopilus balloui* complex are characterized by an adnate hymenophore, depressed around the stipe apex, and occasionally decurrent with a tooth, and with round or angular pores of a smaller diameter.

*Tylopilus pseudoballoui* is the species most similar to *T. aurantiovulpinus* in morphology, but it differs by the smaller pores of the hymenophore. *T. aurantiacus*, *T. rubrotinctus*, and *T. leucomycelinus* have a brighter color with a predominance of red shades. Additionally, there are differences in spore size: *T. aurantiacus* have slightly smaller and more rounded spores, and *T. leucomycelinus* have slightly larger and more rounded spores. *T. fuscatus*, *T. griseolus*, and *T. griseiviridus* are distinguished by the presence of gray shades in the color of the pileus and stem. *T. oradivensis*, *T. pseudoleucomycelinus*, and *T. pygmaeus* have noticeably smaller basidiomata.

#### 3.2.2. Annotated List of the Species of the Boletoid Fungi Recorded for the First Time in Vietnam

1. *Cyanoboletus flavocontextus* L. Fan, N. Mao & T.Y. Zhao, Mycosphere 14(1): 2013–2091 (2023).

**Figure 6:**
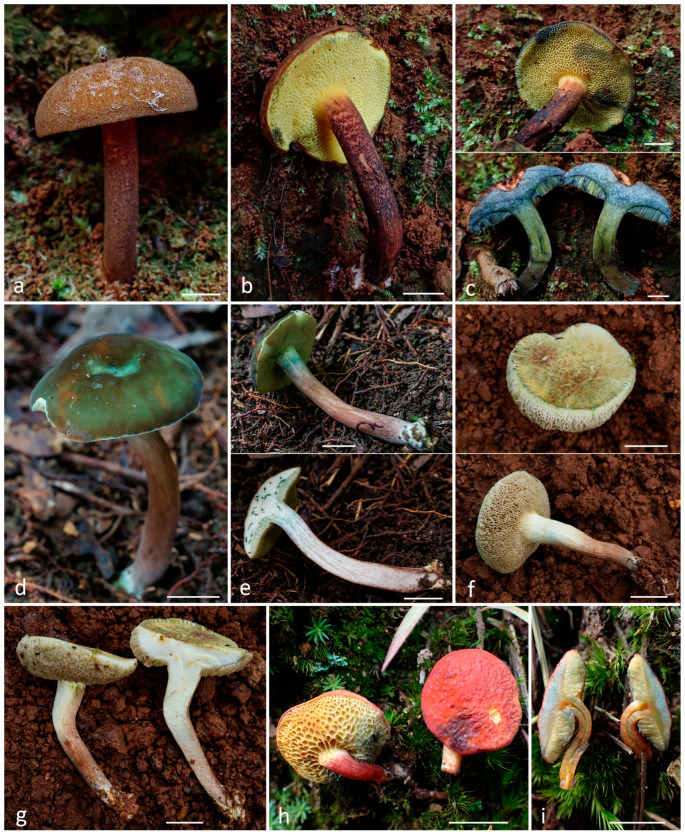
Basidiomata in situ: (**a**–**c**)—*Cyanoboletus flavocontextus* LE F-344052 (48HG23); (**d**,**e**)—*Kgaria virescens* LE F-315591 (261VN16); (**f**,**g**)—*Kgaria virescens* LE F-344056 (138VN23); (**h**,**i**)—*Parvixerocomus pseudoaokii* LE F-344057 (70HG23). Scale bars 1 cm.

*Specimens examined*: Kon Tum Plateau, Gia Lai Province, K’Bang District, Son Lang Commune, Kon Chu Rang Nature Reserve, N 14.47584°, E 108.53719°, 940 m a.s.l., on soil in the tropical lower-mountain deciduous polydominant forest with a predominance of *Lauraceae*, *Burseraceae*, *Myrtaceae*, *Hamamelidaceae*, and *Fagaceae* (*Lithocarpus*, *Quercus*, *Castanopsis*), 23 October 2022, col. O.V. Morozova, LE F-344051 (184VN22). Ibid., N 14.28353°, E 108.32156°, 947 m a.s.l., on loose soil next to roads in middle-mountain evergreen mixed forest with a predominance of *Podocarpaceae* (*Dacrydium elatum*, *Dacrycarpus imbricatus*), *Magnoliaceae*, *Burseraceae* (*Canarium*), and *Myrtaceae* (*Syzygium*), 14 July 2023, col. T.H.G. Pham, LE F-344052 (VRTC 48HG23) (Figure 6a–c).

*Known distribution:* China (holotype), Vietnam.

2. *Kgaria virescens* (Har. Takah. & Taneyama) O.V. Morozova, E.S. Popov, T.H.G. Pham, comb. nov.

MycoBank: MB852384.

*Basionym*: *Boletus virescens* Har. Takah. & Taneyama, in Takahashi, Taneyama, Kobayashi, Oba, Hadano, Hadano, Kurogi & Wada. The fungal flora in southwestern Japan, Agarics and boletes 1: 45 (2016).

*Synonyms*: *Tylopilus virescens* (Har. Takah. & Taneyama) N.K. Zeng, H. Chai & Zhi Q. Liang, in Chai, Liang, Xue, Jiang, Luo, Wang, Wu, Tang, Chen, Hong & Zeng, *MycoKeys* 46: 82 (2019).—*Tylopilus callainus* N.K. Zeng, Zhi Q. Liang & M.S. Su, in Liang, Su, Jiang, Hong & Zeng, *Phytotaxa* 343(3): 271 (2018).

*Specimens examined*: Kon Tum Plateau, Gia Lai Province, K’Bang district, Sơn Lang commune, Kon Chu Rang Nature Reserve, N 14.505156°; E 108.581400°, 1000 a.s.l., on soil in the tropical lower mountain deciduous polydominant forest with a predominance of *Lauraceae*, *Burseraceae*, *Myrtaceae*, *Hamamelidaceae*, and *Fagaceae* (*Lithocarpus*, *Quercus*, *Castanopsis*), 28 May 2016, col. E.S. Popov, LE F-315591 (261VN16) (Figure 6d,e). Ibid., 21 May 2023, col. O.V. Morozova, LE F-344056 (VRTC 138VN23) (Figure 6f,g).

Known distribution: Japan (holotype), China (as *Tylopilus callainus*, *T. virescens*), and Vietnam.

Notes: *Kgaria* was recently described as a new porphyrellus-like genus of *Boletaceae* to accommodate *Tylopilus cyanogranulifer*, a dark brown to dull lilac/violet, or, rarely, nearly black bolete with a series of oxidation reactions progressing from blue to red then nearly black and then to a dark-brown spore deposit. Idiosyncratic blue-green pigment encrustations (cyanogranules) and a similarly colored reaction of the hyphae located on pileus and stipe surfaces are also diagnostic [54]. Morphologically, *Tylopilus virescens* is significantly different from species of *Kgaria*, however resembles them the due to characteristic sea-green discoloration of the context when fresh. Based on the results of the phylogenetical analysis, the taxonomic position of *Tylopilus verescens* should be considered uncertain but close to the genus *Kgaria*. In this case, we suggest new combination, *Kgaria virescens.*

3. *Parvixerocomus pseudoaokii* G. Wu, Kuan Zhao & Zhu L. Yang, in Wu, Zhao, Li, Zeng, Feng, Halling & Yang, Fungal Diversity: 10.1007/s13225-015-0322-0, (12) (2015).

*Specimens examined*: Lam Dong Province, Da Lat city, Tram Hanh Commune, on loose soil on the slopes of drainage ditches next to coffee gardens, N 11.856773°, E 108.546125°, 1500 m a.s.l., 25 October 2023, T.H.G. Pham, LE F-344057 (VRTC 70HG23) (Figure 6h,i).

*Known distribution*: China (holotype), Thailand (KX017303), Vietnam.

4. *Phylloporus hainanensis* N.K. Zeng, L.L. Wu & Zhi Q. Liang, in Xue, Zhang, Xu, Xie, Wu, Wang, Tang, Hao, Zhao, Jiang, Li, Yang, Li, Liang & Zeng, Stud. Mycol. 106: 170 (2023).

**Figure 7:**
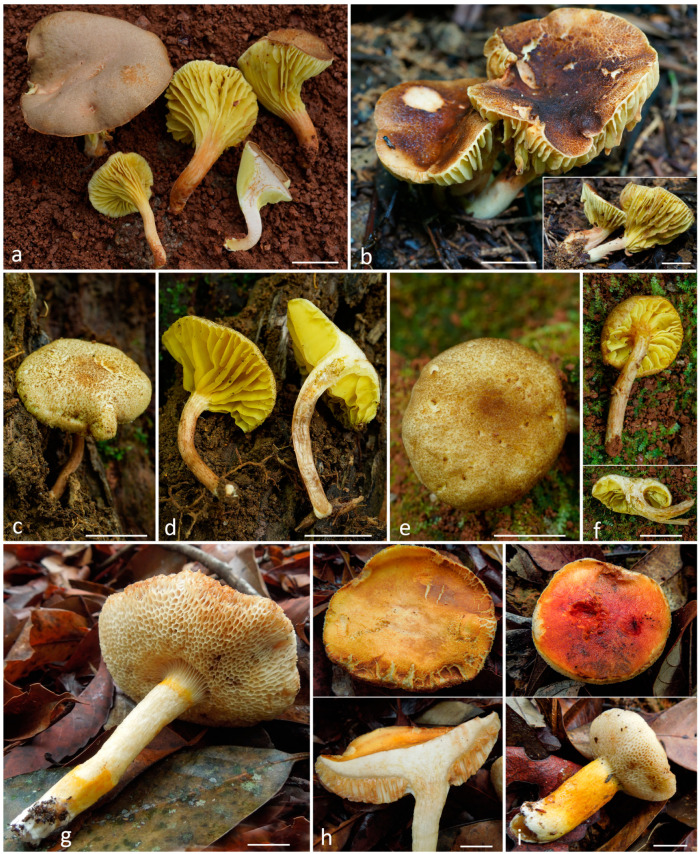
Basidiomata in situ: (**a**)—*Phylloporus hainanensis* 137VN22; (**b**)—*Phylloporus microsquamus* LE 312685 (371VN16); (**c**,**d**)—*Phylloporus nigrisquamus* LE F-344059 (172VN22); (**e**,**f**)—*Phylloporus subbacillisporus* (LE F-344060 (VRTC 193VN22); (**g**–**i**)—*Tylopilus rubrotinctus* LE 312532 (Vn-16-81). Scale bars 1 cm.

*Specimens examined*: Kon Tum Plateau, Gia Lai Province, K’Bang District, Son Lang Commune, Kon Chu Rang Nature Reserve, N 14.47584°, E 108.53719°, 940 m a.s.l., on soil in the tropical lower mountain deciduous polydominant forest with a predominance of *Lauraceae*, *Burseraceae*, *Myrtaceae*, *Hamamelidaceae*, and *Fagaceae* (*Lithocarpus, Quercus, Castanopsis*), 17 May 2023, col. O.V. Morozova and T.H.G. Pham, VRTC 108VN22. Ibid., 20 May 2023, col. O.V. Morozova and T.H.G. Pham, LE F-344058 (VRTC 128VN23). Ibid., col. O.V. Morozova, VRTC 137VN22 (Figure 7a).

*Known distribution*: China (holotype), Vietnam.

5. *Phylloporus microsquamus* N.K. Zeng, L.L. Wu, S. Jiang & Z.Q. Liang, in Wu, Liang, Su, Fan, Zhang, Jiang, Chen, Hao & Zeng, Mycol. Progr. 20(10): 1262 (2021).

*Specimens examined*: Kon Tum Plateau, Kon Plong Protected Forest, north of Kondu Village, valleys of the La and Khe rivers, N 14.73350°, E 108.31292°, 1120 m a.s.l., middle-mountain polydominant forest dominated by *Podocarpaceae*, *Magnoliaceae*, *Myrtaceae*, *Calophyllaceae*, *Elaeocarpaceae*, *Betulaceae*, 10 June 2016, col., O.V. Morozova, LE 312685 (371VN16) (Figure 7b). *Phylloporus* sp. 2 in [9].

*Known distribution*: China (holotype), Vietnam.

6. *Phylloporus nigrisquamus* N.K. Zeng, L.L. Wu & Y.G. Fan, Mycological Progress 20 (10): 1264 (2021).

*Specimens examined:* Dak Nong Province, Dak Glong District, Ta Dung National Park, southeastern macroslope of the ridge of the Ta Dung Mt, south-eastern slope of the Ta Dung Mt, TK 1787, N 11.89603°, E 108.05336°, 1010 m a.s.l., on rotten wood in secondary evergreen broadleaf forest with *Magnoliaceae*, *Fagaceae* (*Castanopsis* sp., *Lithocarpus* sp.), Theaceae and with a significant participation of *Bamboo*, 18 October 2022, T.H.G. Pham, O.V. Morozova, LE F-344059 (VRTC 172VN22) (Figure 7c,d).

*Known distribution*: China (holotype), Vietnam.

7. *Phylloporus subbacillisporus* Raspé, K.D. Hyde & Chuankid, Mycol. Progr. 18 (5): 608 (2019).

*Specimens examined*: Kon Tum Plateau, Gia Lai Province, K’Bang District, Son Lang Commune, Kon Chu Rang Nature Reserve, N 14.47584°, E 108.53719°, 940 m a.s.l., on soil in the tropical lower mountain deciduous polydominant forest with a predominance of *Lauraceae*, *Burseraceae*, *Myrtaceae*, *Hamamelidaceae*, and *Fagaceae* (*Lithocarpus, Quercus, Castanopsis*), 23 October 2022, col. O.V. Morozova and T.H.G. Pham, LE F-344060 (VRTC 193VN22) (Figure 7e,f).

*Known distribution:* China (holotype), Vietnam.

8. *Tylopilus rubrotinctus* Yan C. Li & Zhu L. Yang, The Boletes of China: *Tylopilus* s.l. (Singapore): 339 (2021).

*Specimens examined:* Kon Tum Plateau, Kon Ka Kinh National Park, 14.21988° N, 108.30936° E, 1300 m a.s.l., on the soil in a tropical mountain polydominant forest with the participation of *Myrtaceae*, *Meliaceae*, *Anacardiaceae*, *Fagaceae*, *Theaceae*, 20 May 2016, col. A. V. Alexandrova, LE 312532 (Vn-16-81) (Figure 7g–i), as *Tylopilus* aff. *balloui* in [9].

*Known distribution*: China (holotype), Vietnam.

## 4. Discussion

The morphological examination of the specimens as well as phylogenetic analysis made it possible to reveal two new species for science and raise the question of the taxonomic position of the species known as *Tylopilus virescens*. In all three cases, the taxonomic position of the species in question is ambiguous. The new *Hortiboletus rubroreticulatus* occupies a basal position in the /Hortiboletus clade in the *tef1α* and the ITS trees (together with *Hortiboletus rupicapreus*), in both cases with high statistical support. At the same time, the LSU analysis—which, however, has a lower resolution—does not indicate that the new species belongs to the genus *Hortiboletus*. The heterogeneous structure of the pileipellis, which is not a true polysadoderm, since the hyphae of the pileipellis have different lengths, is also not entirely characteristic for the genus. However, until more data are accumulated, we propose to consider this new species in the genus *Hortiboletus*.

An Important distinguishing featu”e of’the *Tylopilus balloui* species complex is the absence of the bitter taste which is characteristic for typical species of this genus. Taking into account the structural features of the hymenophore discussed above, the lack of a pronounced pink tint in the color of the spores, and also the unstable, near basal position of the species of this group on phylogenetic trees, it can be assumed that this group could also represent a phylogenetic line separate from the *Tylopilus* core. But this issue requires further study.

The taxonomic position of *Tylopilus virescens*, for which we proposed a new combination *Kgaria virescens*, is also controversial and requires the accumulation of additional data, especially on species with a similar type of autooxidation.

As a conclusion, we cannot help but notice that the area of tropical forests currently is drastically declining. Therefore, in Vietnam, in recent decades, much attention has been paid to the conservation of natural landscapes and the protection of biodiversity. There are powerful programs, and the network of natural parks and reserves is expanding. This prevents the continued decline in the area of tropical forests, along with which the species associated with them also disappear, often without even receiving a name. In Central Vietnam, the greatest diversity of fungi of the *Boletaceae* family is found in middle-mountain evergreen broad-leaved forests. They require special attention, study, and careful handling. Studying the fungal diversity of typical tropical forests adds to the knowledge on the macromycetes biota of Vietnam and may be useful in developing conservation measures for these valuable natural areas and the species inhabiting them.

## Figures and Tables

**Table 1:** Specimens and GenBank accession numbers of DNA sequences used in the molecular analyses (Newly generated sequences are in bold).

Species	Location	Voucher Number	Genbank Accession No.			References
			ITS	LSU	*tef1α*	
*Boletus edulis*	Germany	Be3	–	KF030282	–	[39]
*Boletus* sp.	China	HKAS 52639	–	–	KF112195	[37]
*Chalciporus amarellus*	Cyprus	ML901162CA	MH011876	–	–	[40]
*Chalciporus piperatus*	Germany	HKAS 84882	–	KT990562	–	[41]
*Chalciporus piperatus*	USA	Smith-2018	MK573906	–	–	iNaturalist # 17338127
*Chalciporus rubinelloides*	China	HKAS 5736	–	KT990563	–	[41]
*Chiua viridula*	China	HKAS 74928 (T)	–	NG_081278 (KF112483)	–	[37]
*Cyanoboletus flavocontextus*	China	BJTC FM2319-A (T)	OR655149	OR655185	OR659986	[42]
***Cyanoboletus flavocontextus***	**Vietnam**	**LE F-344051 (184VN22)**	**PP317924**	**PP313111**	**PP320320**	**this work**
***Cyanoboletus flavocontextus***	**Vietnam**	**LE F-344052 (48HG23)**	**PP317923**	**–**	**PP320319**	**this work**
*Cyanoboletus mediterraneensis*	Israel	K-M000265124	OM801199	–	–	[43]
*Cyanoboletus pulverulentus*	Italy	MG 628a	KT157055	KT157064	–	[44]
*Cyanoboletus sinopulverulentus* (as *Boletus* sp.)	China	HKAS 59609	–	KF112366	–	[37]
*Cyanoboletus sinopulverulentus* (as *Boletus sinopulverulentus*)	China	HKAS 59609	–	–	KF112193	[41]
*Cyanoboletus sinopulve-rulentus* (as *Boletus sinopulverulentus*)	China	HMAS 266894 (T)	KC579402	–	–	[45]
*Cyanoboletus* sp.	China	OR0257	–	–	MG212587	[46]
*Cyanoboletus* sp.	China	HKAS 59418	–	–	KT990765	[41]
*Fistulinella olivaceoalba*	Vietnam	LE 312004 (T)	–	NG_068853 (MH718396)	–	[24]
*Gyrodon lividus*	Germany	REG Gl1	–	–	GU187701	[47]
*Gyroporus castaneus* (as *Gyroporus* sp.)	China	HKAS 63505	–	KF112476	–	[37]
*Gyroporus castaneus*	USA	JMP0028	EU819468	–	–	[48]
*Gyroporus cyanescens*	USA	MB 05-001	–	EU718138	FJ536672	[49]
*Gyroporus cyanescens*	USA	NAMA190	EU819495	–	–	[48]
*Harrya subalpina*	China	HKAS 90194	–	KT990700	–	[41]
*Hortiboletus amygdalinus*	China	HKAS54166 (T)	–	NG_059622 (KT990581)	KT990777	[41]
*Hortiboletus amygdalinus*	Korea	NIBRFG0000502792	MW578955	–	–	[50]
*Hortiboletus arduinus*	China	FHMU 3323 (T)	–	NG_075378 (MT646432)	–	[51]
***Hortiboletus bubalinus***	**Russia**	**LE F-315839**	**PP317928**	**PP313106**	**PP320315**	**this work**
*Hortiboletus indorubellus*	India	DC 14-002 (T)	NR_154076 (KT319647)	–	–	[52]
*Hortiboletus kohistanensis* (as *Xerocomus* sp.)	Pakistan	AST48 (LAH35327, T)	MG988192	MG988187	–	[53]
*Hortiboletus napaeus*	China	FHMU3326 (T)	NR_184923 (MT646440)	NG_075379 (MT646433)	–	[51]
*Hortiboletus* cf. *rubellus (=Xerocomellus* cf. *rubellus)*	USA	MB03–033	–	KF030294	KF030419	[39]
*Hortiboletus* sp.	Germany	KR-M-0044799	MT006029			Genbank
***Hortiboletus rubroreticulatus***	**Vietnam**	**LE F-344053 (T)**	**PP317926**	**PP313108**	**PP320317**	**this work**
***Hortiboletus rubroreticulatus***	**Vietnam**	**LE F-344054**	**PP317927**	**PP313107**	**PP320316**	**this work**
*Hortiboletus rufosquamosus*	China	BJTC FM2649	–	–	OR659988	[42]
*Hortiboletus rupicapreus*	Vietnam	LE 312677 (T)	MW784161	MW760391	MZ424894	[29]
*Hortiboletus rupicapreus*	Vietnam	LE 312678	MW784162	–	MZ424893	[29]
***Hortiboletus rupicapreus***	**Vietnam**	**LE F-344055 (130VN22)**	**PP317925**	**–**	**–**	**this work**
*Hortiboletus subpaludosus*	China	HKAS68158	-	-	KT990779	[41]
*Hortiboletus tomentosus*	China	BJTC FM2289-A (T)	–	–	OR659994	[42]
*Kgaria cyanogranulifera*	Australia	NY1115381 (REH9189)	–	OR063859	OR232707	[54]
*Kgaria cyanogranulifera*	Australia	NY1194100 (REH9359)	–	OR063862	OR232708	[54]
*Kgaria similis*	Australia	NY1193839 (REH9406)	–	OR063867	OR113660	[54]
*Kgaria similis*	Australia	NY1193974 (REH9031, T)	–	OR063865	–	[54]
***Kgaria virescens***	**Vietnam**	**LE F-344056 (138VN23)**	**PP317930**	**PP313104**	**PP320322**	**this work**
***Kgaria virescens***	**Vietnam**	**LE F-315591 (261VN16)**	**–**	**–**	**PP320321**	**this work**
*Lanmaoa asiatica*	China	HKAS 63603	–	–	KM605153	[55]
*Parvixerocomus pseudoaokii*	China	HKAS 80480 (T)	–	NG_059543 (KP658468)	–	[55]
*Parvixerocomus pseudoaokii*	Thailand	CMU58-ST-0504	KX017303	–	–	Genbank
*Parvixerocomus pseudoaokii*	China	OR0155	–	–	MG212597	[46]
***Parvixerocomus pseudoaokii***	**Vietnam**	**LE F-344057 (70HG23)**	**PP317937**	**PP313110**	**–**	**this work**
*Parvixerocomus matheranensis*	India	AMH 9976 (T)	–	–	MT140890	[56]
*Phylloporus hainanensis*	China	Zeng2724 (FHMU1718, T)	MW588632	MW588581	–	[57]
*Phylloporus hainanensis*	China	N.K. Zeng 4984 (FHMU5550)	–	–	ON868501	[58]
***Phylloporus hainanensis***	**Vietnam**	**LE F-344058 (128VN23)**	**PP317934**	**PP313115**	**PP320324**	**this work**
***Phylloporus hainanensis***	**Vietnam**	**137VN23**	**PP317935**	**–**	**–**	**this work**
***Phylloporus hainanensis***	**Vietnam**	**108VN23**	**PP317933**	**–**	**–**	**this work**
*Phylloporus luxiensis*	Vietnam	LE 315622	–	–	MZ424877	[9]
*Phylloporus microsquamus*	China	FHMU 1678 (T)	NR_175702 (MW588648)	NG_088206 (MW588599)	–	[57]
*Phylloporus microsquamus*	China	OR0258	–	–	MH580809	[59]
*Phylloporus microsquamus*	Vietnam	LE 312685	MZ451354	**PP313113**	–	[9] as *Phyllo-porus* sp.)
*Phylloporus microsquamus*	Vietnam	LE 312684	–	–	MZ424892	[9]
*Phylloporus nigrisquamus*	Thailand	MAN131	JQ003649	–	–	[57]
*Phylloporus nigrisquamus*	China	Y.G. Fan 2819 (FHMU3271, T)	–	NG_088205 (MW588590)	–	[57]
***Phylloporus nigrisquamus***	**Vietnam**	**LE F-344059 (172VN22)**	**PP317936**	**PP313114**	**PP320325**	**this work**
*Phylloporus subbacillisporus*	Thailand	OR0989	MH686277	–	–	[59]
*Phylloporus subbacillisporus*	China	OR0436 (HMAS 279879, T)	NR_173793 (MH686274)		MH580812	[59]
*Phylloporus subbacillisporus*	China	HKAS 74682	–	JQ967230	–	[60]
***Phylloporus subbacillisporus***	**Vietnam**	**LE F-344060 (193VN22)**	**PP317932**	**PP313116**	**PP320326**	**this work**
*Porphyrellus porphyrosporus*	China	HKAS 49182 (Ge687)	–	KT990544	–	[41,61]
*Porphyrellus porphyrosporus* (as *Tylopilus porphyrosporus*)	China	HKAS 76671	–	–	KF112243	[37]
*Pseudoaustroboletus valens*	China	HKAS 82644 (LF690)	–	MT154749	–	[61]
*Pulveroboletus flaviscabrosus*	China	HKAS83190 (T)		KX453821		[62]
*Pulveroboletus subrufus*	China	HKAS84926	–	KX453836	–	[62]
*Retiboletus griseus*	China	HKAS:63590	–	KF112417	–	[37]
*Tylopilus aurantiacus*	China	HKAS59700 (Li1952, T)	–	NG_081276 (KF112458)	KF112223	[61]
***Tylopilus aurantiovulpinus***	**Vietnam**	**LE F-344061 (T)**	**PP317938**	**PP313109**	**PP320323**	**this work**
*Tylopilus aurantiovulpi-nus* (as *T.* aff. *balloui*)	Vietnam	LE 312700	MZ451358	–	MZ424886	[9]
*Tylopilus balloui*	USA	NY, Halling 8292	–	EU430734	–	[63]
*Tylopilus balloui*	Thailand	CMU51-SL-39	KX017306	–	–	Genbank
*Tylopilus callainus*	China	FHMU N.K.Zeng 1459 (T)	MG365901	MG365896	–	[64]
*Tylopilus callainus*	China	FHMU N.K.Zeng 1464	–	–	MG365904	[64]
*Tylopilus felleus*	Sweden	AT2001011	UDB000680	JQ326993	JQ327015	UNITE DB; [65]
*Tylopilus leucomycelinus*	Belize	NY00796119	–	EU430735	–	[63]
*Tylopilus microsporus*	China	HMAS 84730 (T)	NR_137924 (KM975485)	–	–	[44]
*Tylopilus otsuensis*	China	HKAS 53401	–	KF112449	–	[37]
*Tylopilus pseudoballoui*	India	DC 17-35 (T)	MG799324	MG799325	–	[66]
*Tylopilus pseudoballoui* (as *T. balloui*)	China	HKAS51151 (Li714)	–	–	MW165265	[61]
*Tylopilus pseudoleucomycelinus*	Mexico	MEXU: HO 30115	–	OQ940037	–	[67]
*Tylopilus rubrotinctus*	China	HKAS 80684 (KK259, T)	–	NG_088129 (MT154733)	MW165264	[61]
*Tylopilus rubrotinctus* (as *T*. aff. *balloui*)	Vietnam	LE 312532	MZ451357	–	MZ424883	[9]
*Veloporphyrellus vulpinus*	Vietnam	LE 315544 (T)	–	MN511170	–	[25]
*Xanthoconium sinense*	China	HKAS77758 (T)	–	NG_059630 (KT990665)	–	[41]
*Xerocomellus bolinii*	USA	JAB_95	–	–	MW737491	[68]
*Xerocomellus chrysenteron*	Spain	AH38968	KU355473	–	–	Genbank
***Xerocomellus cisalpinus***	**Russia**	**LE F-343575**	**PP317929**	**–**	**PP320318**	**this work**
*Xerocomellus cisalpinus*	Finland	AT2005034	–	–	KF030417	[39]
***Xerocomellus cisalpinus***	**Russia**	**LE 315834**	**–**	**PP313105**	**–**	**this work**
*Xerocomellus poederi*	Spain	AH 44050 (T)	NR_155971 (KU355475)	NG_060000 (KU355488)	–	[69]
*Xerocomellus sarnarii*	Italy	MCVE 28577 (T)	NR_138006 (KT271749)	–	–	[70]
*Xerocomus ferrugineus*	Sweden	AT1999098	DQ066398	–	–	[71]
*Xerocomus ferrugineus*	China	BJTC FM1245	–	OR655219	OR660018	[42]
*Xerocomus magniporus*	China	HKAS 59820	–	–	JQ967195	[60]
***Xerocomus* sp.**	**Vietnam**	**LE F-344062 (** **165VN22)**	**PP317931**	**PP313112**	**PP320327**	**this work**
*Xerocomus squamulosus*	New Zealand	PDD 101777	OP141473	–	–	Genbank
*Xerocomus squamulosus*	New Zealand	JAC10883	–	OP141507	–	Genbank
*Xerocomus subparvus*	Vietnam	LE315595	MT893600	–	–	[3]
*Xerocomus subsplendidus*	China	HFJAU12011	–	–	OQ162214	[72]
*Xerocomus subtomentosus*	Sweden	AT2002025a	DQ066361	–	–	[71]

## Data Availability

The DNA sequence data obtained from this study have been deposited in GenBank NCBI (https://www.ncbi.nlm.nih.gov/genbank/, accessed on 5 February 2024).
